# A Concept of Cross-Ferroic Plasma Turbulence

**DOI:** 10.1038/srep22189

**Published:** 2016-02-26

**Authors:** S. Inagaki, T. Kobayashi, Y. Kosuga, S.-I. Itoh, T. Mitsuzono, Y. Nagashima, H. Arakawa, T. Yamada, Y. Miwa, N. Kasuya, M. Sasaki, M. Lesur, A. Fujisawa, K. Itoh

**Affiliations:** 1 Research Institute for Applied Mechanics, Kyushu University, 6-1 Kasuga-Koen, Kasuga-city, Fukuoka 816-8580, Japan; 2 Research Center for Plasma Turbulence, Kyushu University, 6-1 Kasuga-Koen, Kasuga-city, Fukuoka 816-8580 Japan; 3 National Institute for Fusion Science, 322-6 Oroshi-cho, Toki-city, Gifu 509-5292, Japan; 4 Institute for Advanced Study, Kyushu University, 6-10-1 Hakozaki, Higashi-ku, 812-8581, Fukuoka Japan; 5 Interdisciplinary Graduate School of Engineering Sciences, Kyushu University, 6-1 Kasuga-Koen, Kasuga-city, Fukuoka 816-8580, Japan; 6 Teikyo University, 6-22 Misaki-machi, Omuta-city, Fukuoka 836-8505, Japan; 7 Faculty of Arts and Science, Kyushu University, 744 Motooka, Nishi-ku, Fukuoka 819-0395, Japan

## Abstract

The variety of scalar and vector fields in laboratory and nature plasmas is formed by plasma turbulence. Drift-wave fluctuations, driven by density gradients in magnetized plasmas, are known to relax the density gradient while they can generate flows. On the other hand, the sheared flow in the direction of magnetic fields causes Kelvin-Helmholtz type instabilities, which mix particle and momentum. These different types of fluctuations coexist in laboratory and nature, so that the multiple mechanisms for structural formation exist in extremely non-equilibrium plasmas. Here we report the discovery of a new order in plasma turbulence, in which chained structure formation is realized by cross-interaction between inhomogeneities of scalar and vector fields. The concept of *cross-ferroic turbulence* is developed, and the causal relation in the multiple mechanisms behind structural formation is identified, by measuring the relaxation rate and dissipation power caused by the complex turbulence-driven flux.

“Panta Rhei - everything flows and nothing lasts (Heraclitus)”, is true in our universe, because one structure that decays is generated after another. The variety of scalar and vector fields in nature is formed by plasma turbulence^1^. Drift-wave fluctuations^2^, which are excited by density gradient in magnetized plasmas, play important roles in turbulent structure formation in laboratory plasmas (such as for fusion-oriented research). Drift waves drive cross-field flux to generate, e.g. zonal flows and zonal magnetic fields, which circumnavigate the main magnetic field^1^,^3^,^4^. On the other hand, the sheared flow in the direction of magnetic fields is predicted to cause Kelvin-Helmholtz type instability^5^ (D’Angelo mode^6^). This instability is considered to be a key element in understanding the turbulent transport in space plasmas. Both drift wave and D’Angelo mode fluctuations induce cross-field fluxes of particle and momentum, and the interferences and competition between gradients of different fields (density, azimuthal and axial velocities) take place^1^,^2^,^5^,^7^,^8^. (Concerning the flow-shear driven mode, although it was identified experimentally^5^,^9^,^10^, direct interference between particle and density fluxes associated with this mode has not been measured.) Thus, there is a central problem, i.e., what would happen if both the pressure-gradient driven turbulence and flow-shear-driven turbulence coexist simultaneously? How does it impact structural formation in extremely non-equilibrium plasmas? Here we report the discovery of a new order in cross-ferroic turbulence, in which chained structure formation is realized by cross-interaction between different inhomogeneities of scalar and vector fields. Drift-wave fluctuations cause cross-field flux of momentum, which enhances the velocity gradient. The enhanced velocity gradient excites Kelvin-Helmholtz type instability, which causes an up-hill flux of particles in the strong-velocity-shear region. The causal relation in the competing structure formations is identified, by measuring the relaxation rate and dissipation power in the complex turbulence-driven flux.

## Results

The interference and competing formation of large-scale orders are experimentally identified on the linear magnetized plasma in PANTA (Plasma Assembly for Nonlinear Turbulence Analysis)^11^. A homogeneous axial (*z*) magnetic field of *B* = 0.09 T confines plasma in the radial (*r*) direction. The azimuthal (*θ*) direction, which corresponds to the electron diamagnetic direction, is defined as right-hand direction of *z*. A helicon plasma source is located at one side of a vessel, where a double loop antenna on a quartz tube (radius 5 cm, located at *z* = 0 m) is installed. Argon gas at 0.1 Pa is fed in the quartz tube and is ionized by 2.7 kW of 7 MHz rf waves from an antenna on the tube. On the other side of the vessel, the plasma is terminated by an endplate (stainless steel). This boundary condition (source at one end and metal plate at the other end) induces a plasma flow and weak gradient of electron pressure in the direction of the magnetic field, and determines the axial mode structure of the drift wave. In the region of *z* < 2.5 m, the cross-field transport by turbulence is dominant loss channel of plasma, and the plasma weakly varies along the field line. Typical plasma parameters are as follows: plasma radius *a* ~ 5 cm, plasma length *L* ~ 4 m, electron density 〈*n*_e_〉 ~ 1 × 10^19^ m^−3^, electron temperature 〈*T*_e_〉 ~ 3 eV, and ion temperature 〈*T*_i_〉 ~ 0.3 eV, where 〈〉 denotes long time average. A 4-tips probe is used to measure scalar-fields (*n*_e_, *T*_e_) and flow vector-fields (*V*_*r*_* , V_θ_*, *V*_*z*_) (see ‘Methods’). The fluctuation component of a general quantity *A* is defined as 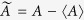. The axial location of the probe can be translocated to *z* = 0.625, 1.125, 1.625, 2.625, 3.125, 3.375 m in a shot by shot manner.

**Figure 1:**
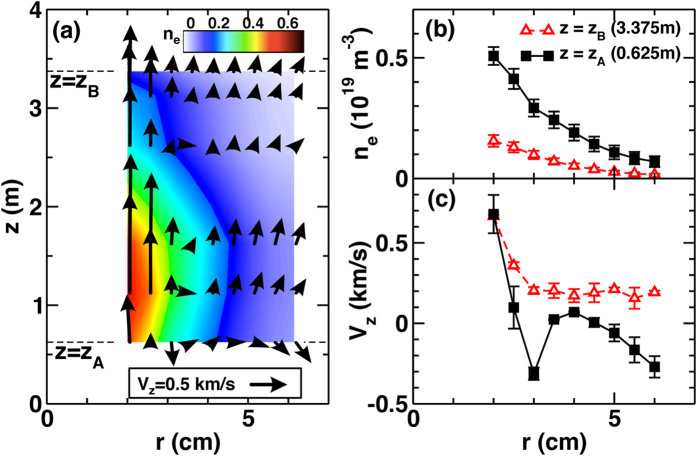
Structure formation in magnetized plasma. Contour plot of density and projected view of flow vector field on the *r*-*z* plane (**a**). Radial profiles of density (**b**) and axial flow velocity (**c**) at two different axial locations (*z*_A_, *z*_B_).

Figure 1(a) illustrates the density profile and time-averaged plasma flow (arrows) on the *r*-*z* plane. Figure 1(b,c) show radial profiles of density and axial flow at two axial locations, *z*_A_ = 0.625 m and *z*_B_ = 3.375 m. A striking observation is that the particle flux is inward (up-gradient) and the axial flow is reversed in some area (e.g., near center at *z* = *z*_A_).

**Figure 2:**
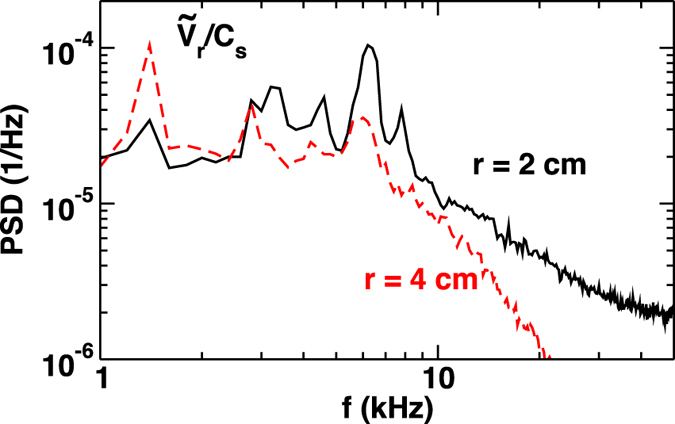
Power spectrum density of radial flow velocity at the inner (*r* = 2 cm) and outer (*r* = 4 cm) regions of plasma column.

These complex behaviors of flow are induced by fluctuations. The spatiotemporal structure of fluctuations in the PANTA plasma is measured by a 64-channel probe array^12^. Figure 2 indicates that the frequencies of the strongest coherent peaks in the power spectrum of 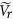 at *r* = 4 cm are 2.8 kHz (which satisfies the dispersion relation of drift waves) and 1.2 kHz (a mediator of the streamer^12^). In contrast, at *r* = 2 cm, the peak of spectrum appears at 6.5 kHz, and the higher frequency broadband modes are enhanced compared to that at *r* = 4 cm. The peak at 6.5 kHz is considered to be a D’Angelo mode (the details are discussed later).

**Figure 3:**
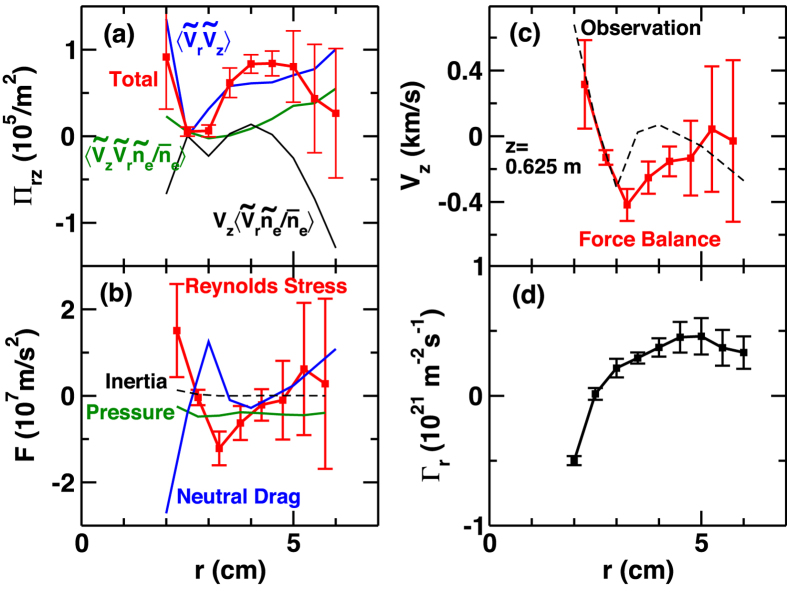
Radial profiles (at *z* = *z*_A_) of (**a**) Reynolds stress, (**b**) force density, (**c**) axial flow velocity and (**d**) radial particle flux.

The momentum and particle transport driven by fluctuations are shown in Fig. 3 (at *z* = *z*_A_). Here Reynolds stress is calculated as^13^,


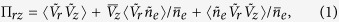


where 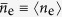. The first, second and third terms are shown in Fig. 3(a). The total Reynolds stress has a dip around *r* = 2.5–3 cm. Thus the opposite of the divergence of Reynolds stress, −(*r*^−1^∂(*r*Π_*rz*_)/∂*r*), i.e. the driving force of the flow, is negative in the region of *r* = 3–4 cm, where the inversion of axial flow is observed. The four terms in the force balance equation, 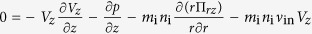, (where *p* is total pressure, *m*_i_ is the ion mass, ν_in_ is the ion-neutral collision frequency) are shown in Fig. 3(b). The inertia term *V*_*z*_∂*V*_*z*_/∂*z* is negligibly small compared to the other terms in the region of measurement (Fig. 1(a)). The parallel pressure gradient term is also small at this location (*z* = *z*_A_). Hence the dominant drive of the flow is the Reynolds stress term, which balances with neutral drag to determine *V*_z_. We substitute a neutral drag coefficient ν_in_ = 40 kHz, which is assumed to be constant over the plasma column and is evaluated based on neutral pressure measurement with an ionization gauge^14^. The *V*_z_ estimated by force balance (dotted line in Fig. 3(c)) is consistent with the observed steep gradient and inversion of flow near the center (r = 2 ~ 3 cm). This inversion of axial flow in Fig. 1(c) is caused by the Reynolds stress of drift-wave fluctuations. Drift-waves are excited by radial density gradient and drive an outward particle flux in the outer portion of plasma (*r* > 2.5 cm) as shown in Fig. 3(d). The outward flux relaxes the density gradient. On the other hand, an inward particle flux is generated at *r* = 2 cm by D’Angelo mode, which is excited by the steep gradient of velocity near the center. The inward flux steepens the density profile in the central region. This is a *cross-ferroic* structure formation via plasma turbulence, in the sense that different fields (density and velocity) and corresponding fluxes (particle and momentum) cross-interact.

**Figure 4:**
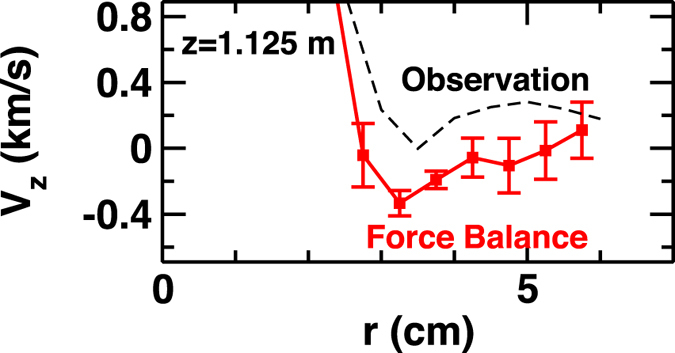
Radial profiles of calculated- and observed-axial flow velocity at z = 1.125 m.

The force balance is also evaluated at z = 1.123 m. The Reynolds force plays a dominant role again in the flow-drive terms. In the region (z < 2 m), the turbulence driven-*radial* flux is dominant over the loss along the field line as the relaxation channel of particle/mass. The calculation is compared with observations as shown in Fig. 4. There is small discrepancy (off-set) in the magnitude between the observation and calculation. This originates from uncertainty of the estimation of density profile of neutral particle in the model. Although there is uncertainty in the evaluation of neutral drag term, the calculated flow is consistent with the observation qualitatively in that: 1) Strong axial flow and shear layer is formed in the central region. 2) The dip of the axial flow is located at around *r* = 3 cm. We thus stress that the picture of structure formation by turbulence holds in wider region along the field line.

This chained structure formation can be understood quantitatively by analyzing the stability condition of D’Angelo^6^. The instability condition is obtained via a fluid model as 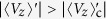, where


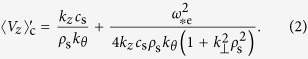


**Figure 5:**
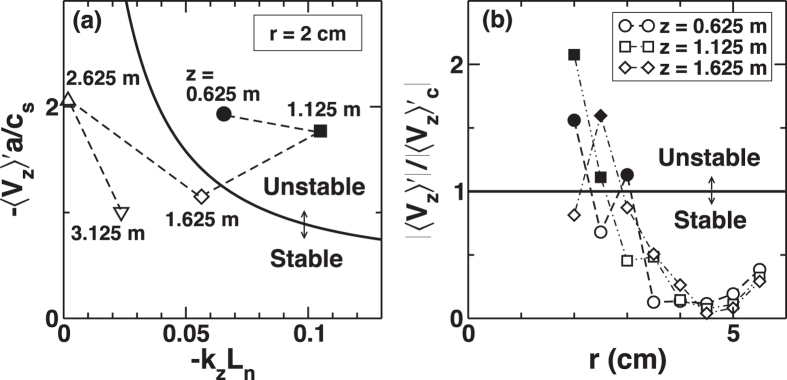
Stability diagram for D’Angelo mode. Diagram on the *k*_*z*_*L*_n_ −〈*V*_*z*_〉′*a*/*c*_s_ plane where *L*_n_ is the density gradient scale length (**a**). Radial profiles of normalized axial flow gradient at three different *z*-locations (**b**). Solid lines denote the stability boundary, above which the theoretical model predicts instability. Symbols denote experimental observations.

Here, *c*_s_ is the ion sound speed, *ρ*_s_ is the ion sound Larmor radius, ω_*e_ is the drift wave frequency, 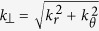, and 〈*V*_*z*_〉′ denotes ∂〈*V*_*z*_〉/∂*r*^15^. Figure 5 shows the stability diagram for experimental parameters, 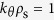. D’Angelo mode can be unstable in inner (2–3 cm) regions close to the source region (*z* = 0.625, 1.125 m). When the velocity profile is monotonic and the gradient is weak (as is the case at *z* = 3.375 m in Fig. 1(c)), the D’Angelo mode is linearly stable. When the drift-wave and D’Angelo mode fluctuations coexist, the interference of particle and momentum fluxes takes place. The result of quasi-linear theory for particle and momentum fluxes is:


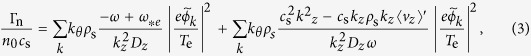



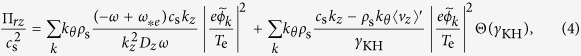


where ϕ_*k*_ is the potential fluctuation of both modes and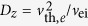, Θ is the step function, and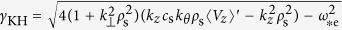 is the growth rate of the D’Angelo mode. The first terms in the RHS of Eq. (3) and (4) are the effects from density gradient, and the second terms are driven by 〈*V*_*z*_〉′. The second term in the RHS of Eq. (3) is always negative (i.e., the particle flux is up-hill) for unstable D’Angelo mode 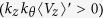. Thus, the net particle flux can be inward when this term becomes dominant, i.e. parallel flow shear dominates the fluctuations. In turn, while releasing free energy stored in ∇*n*_e_, the drift waves exert Reynolds stress to drive macroscopic flows. The momentum flow by ∇*n*_e_-driven drift-wave turbulence has been discussed in the literature^13^,^16^,^17^. However, the present observation indicates, for the first time, interferences between fluxes of particle and axial momentum. We found a chained interaction in the observed turbulence. That is, the drift wave induces momentum flux, which reverses the flow direction. The enhanced velocity gradient excites the D’Angelo mode, which is stable without flow reversal (for the present parameters). D’Angelo mode causes an up-hill particle flux near the axis.

## Discussion

The primary origin in this chained structural formation is examined via the rate of dissipations^18^,^19^. Each energy relaxation rate *W*_j_ associated with the *j*-th turbulence-driven flow *V*_*j*_ (*j* = *r*, *θ*, *z*), is evaluated from the volume integral of *W*_*j*_ = *F*_*j*_ *V*_*j*_, where *F* is the force density, **F** = (−∂*p*_e_/∂*r*, −*n*_i_*m*_i_∂(*r*Π_*r*θ_)/*r*∂*r*, −*n*_i_*m*_i_∂(*r*Π_*rz*_)/*r*∂*r*). The energy relaxation rates (*W*_*r*_, *W*_θ_, *W*_*z*_) are calculated as (76 W, 0.16 W, 11 W), respectively. These values are obtained by integrating the power density over a volume (0.625 m < *z* < 3.375 m, 2 cm < *r* < 6 cm, 0 < *θ* < 2π). The dissipation power of fluctuation energy, *W*_turb,_ is evaluated by the volume integral of 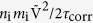. Here the correlation time of turbulence is estimated as τ_corr_ ~ 1 ms from Fig. 2, and *W*_turb_ is estimated as 4.1 W. From these evaluations, we find that 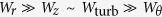. The power *W*_*r*_ is dominant and thus the main relaxation channel of turbulent processes is the decay of density profile via radial flow.

In conclusion, in *cross-ferroic turbulence*, we analyzed the turbulent structural formation of scalar and vector fields of extremely nonequilibrium magnetized plasma. The results show elements in *cross-ferroic turbulence*: (i) Scalar-fields and vector-fields spontaneously produce global-scale orders. (ii) Both fields interfere with each other, yielding a chained structure in a selected region of plasma. (iii) The dominant cause can be detected by evaluating the non-linear relaxation rate. Namely, the drift-wave fluctuations drive momentum transport, which generates the inversion and strong shear of axial flow in the central region. The enhanced gradient of flow velocity, in turn, drives the D’Angelo mode, which causes particle pinch and collimates the plasma density in the central region.

## Methods

### 4-tips probe measurement

Two tips are aligned in the axial direction, to measure the axial velocity, 〈*V*_z_〉 and 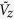, with Mach probe technique. The other two are coordinated in the azimuthal direction for radial velocity measurement (〈*V*_r_〉 and 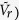, through the difference of floating potentials, *ϕ*_f_. The axial flow velocity is evaluated as *V*_*z*_* = c*_s_(2*T*_e_*/T*_i_)^1/2^(*I*_u_ − *I*_d_)/(*I*_u_* + I*_d_)^20^, where *I*_u_ and *I*_d_ denote ion saturation currents in the upstream and downstream side, respectively. An alternative model to evaluate the flow velocity yields identical results. The upstream tip and downstream tip are exchanged in a shot-by-shot manner to balance the small individual variability of tips. The error bars in the Reynolds force term in Fig. 3 are originated from the asymmetry of the tips. Double-probe method is also applied by using a pair of tips, where 〈*T*_e_〉 and 〈*n*_e_〉 are evaluated by using similar discharges. The ion temperature of 〈*T*_i_ 〉 ~ 0.3 eV is measured by use of an ion sensitive probe. The long time-averaged potential 〈*ϕ*〉 is estimated from the floating potential 〈*ϕ*_f_〉, while considering the impact of 〈*T*_e_〉. Relative density fluctuations are provided by relative ion saturation current fluctuations, as 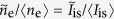. The temperature fluctuation amplitude 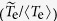 of the drift-wave is evaluated as ~10% of 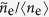
and 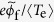
by using a triple-probe method in PANTA^21^. In this study, the temperature fluctuation is not monitored but taken into account in the measurement error for evaluating the level of density and flow velocity fluctuations. The measurement errors by temperature fluctuation are estimated to be at most 5% for 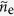 and 50% for 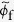, respectively. The probe head is movable (*r* > 2 cm), without disturbing plasma turbulence.

## Additional Information

**How to cite this article**: Inagaki, S. *et al.* A Concept of Cross-Ferroic Plasma Turbulence. *Sci. Rep.* **6**, 22189; doi: 10.1038/srep22189 (2016).

## References

[b1] Diamond P. H., Itoh S.-I., Itoh K. & Hahm T. S. Zonal flows in plasma—a review. Plasma Phys. Control. Fusion 47, R35 (2005).

[b2] Kadomtsev B. B. Plasma Turbulence Academic, New York, 78–106 (1965).

[b3] Fujisawa A. *et al.* Identification of Zonal Flows in a Toroidal Plasma. Phys. Rev. Lett. 93, 165001 (2004). 15524996 10.1103/PhysRevLett.93.165002

[b4] Fujisawa A. *et al.* Experimental Evidence of a Zonal Magnetic Field in a Toroidal Plasma. Phys. Rev. Lett. 98, 165001 (2007). 17501426 10.1103/PhysRevLett.98.165001

[b5] Amatucci W. E. Inhomogeneous plasma flows: A review of *in situ* observations and laboratory experiments. J. Geophy. Res. 104, 14481 (1999).

[b6] D’Angelo N. Kelvin—Helmholtz Instability in a Fully Ionized Plasma in a Magnetic Field. Phys. Fluids 8, 1748 (1965).

[b7] Bai L., Fukuyama A. & Uchida M. Flow shear stabilization of a hybrid dissipative trapped electron-ion temperature gradient mode in tokamaks. Plasma Phys. Control. Fusion 40, 785 (1998).

[b8] Hasegawa H. *et al.* Transport of solar wind into Earth’s magnetosphere through rolled-up Kelvin–Helmholtz vortices. Nature 430, 755 (2004). 15306802 10.1038/nature02799

[b9] Agrimson E. D’Angelo N. & Merlino R. L. Excitation of Ion-Acoustic-Like Waves by Subcritical Currents in a Plasma Having Equal Electron and Ion Temperatures. Phys. Rev. Lett. 86, 5282 (2001). 11384478 10.1103/PhysRevLett.86.5282

[b10] Kaneko T., Tsunoyama H. & Hatakeyama R. Drift-Wave Instability Excited by Field-Aligned Ion Flow Velocity Shear in the Absence of Electron Current. Phys. Rev. Lett 90, 125001 (2003). 12688879 10.1103/PhysRevLett.90.125001

[b11] Yamada T. *et al.* End plate biasing experiments in linear magnetized plasmas. Nucl. Fusion 54, 114010 (2014).

[b12] Yamada T. *et al.* Anatomy of plasma turbulence. Nature Phys. 4, 721 (2008).

[b13] Diamond P. H. *et al.* An overview of intrinsic torque and momentum transport bifurcations in toroidal plasmas. Nucl. Fusion 53, 104019 (2013).

[b14] Nagashima Y. *et al.* Observation of the parametric-modulational instability between the drift-wave fluctuation and azimuthally symmetric sheared radial electric field oscillation in a cylindrical laboratory plasma. Phys. Plasmas 16, 020706 (2009).

[b15] Kosuga Y., Itoh S.-I. & Itoh K. Density peaking by parallel flow shear driven instability in PANTA. Plasma Fusion Res. 10, 3401024 (2015).

[b16] Mattor N. & Diamond P. H. Momentum and thermal transport in neutral‐beam‐heated tokamaks. Phys. Fluids 31, 1180 (1988).

[b17] Angioni C. *et al.* Intrinsic Toroidal Rotation, Density Peaking, and Turbulence Regimes in the Core of Tokamak Plasmas. Phys. Rev. Lett. 107, 215003 (2011). 22181890 10.1103/PhysRevLett.107.215003

[b18] Itoh K. & Itoh S.-I. Entropy Production and Inward Heat Pinch of Plasma. J. Phys. Soc. Jpn. 65, 468 (1996).

[b19] Kosuga Y. *et al.* On the efficiency of intrinsic rotation generation in tokamaks. Phys. Plasmas 17, 102313 (2010).

[b20] Hudis M. & Lidsky L. M. Directional Langmuir Probe, J. Appl. Phys. 41, 5011 (1970).

[b21] Kawashima K. *et al.* Evaluation of Electron Temperature Fluctuations Using Two Different Probe Techniques in Plasma Assembly for Nonlinear Turbulence Analysis (PANTA). Plasma Fusion Res. 6, 2406118 (2011).

